# Pathological pigmentation in cardiac tissues of Atlantic salmon (*Salmo salar* L.) with cardiomyopathy syndrome

**DOI:** 10.1186/1297-9716-44-107

**Published:** 2013-11-13

**Authors:** Hilde AS Fagerland, Lars Austbø, Camilla Fritsvold, Marta Alarcon, Espen Rimstad, Knut Falk, Torunn Taksdal, Erling O Koppang

**Affiliations:** 1 Section of Anatomy and Pathology, Department of Basic Science and Aquatic Medicine, Norwegian School of Veterinary Science, 0033 Oslo, Norway; 2 Section of Genetics, Department of Basic Science and Aquatic Medicine, Norwegian School of Veterinary Science, 0033 Oslo, Norway; 3 Norwegian Veterinary Institute, Pb 750 Sentrum, N-0106 Oslo, Norway; 4 Section of Microbiology, Immunology and Parasitology, Department Food Safety and Infection Biology, Norwegian School of Veterinary Science, 0033 Oslo, Norway

## Abstract

It is widely accepted that melanin formation may play an immunologic role in invertebrates and ectothermic vertebrates. In farmed Atlantic salmon, cardiomyopathy syndrome (CMS) is a common viral disease associated with severe cardiac inflammation that may be accompanied by heavy melanisation of the heart. By the use of histology, laser capture microdissection and transcription analysis of tyrosinase genes, we here show that this melanisation is linked to *de novo* melanogenesis by melanomacrophages, suggesting an active part in the inflammatory reaction. No general systemic activation of the extracutaneous pigmentary system in response to viral infections with affinity to the heart was observed.

## Introduction, methods and results

Cardiomyopathy syndrome (CMS) is a severe disease that affects farmed Atlantic salmon (*Salmo salar* L.). The piscine myocarditis virus (PMCV) is the proposed causative agent [1] and the pathological changes are characterized by moderate to severe inflammation of the heart, mostly limited to the endocardium and spongy myocardium in the atrium and ventricle [2,3]. Abundant melanin deposits can be found located in severe myocardial lesions of CMS [3], but the reason of such melanisation is enigmatic.

Melanin may occur at sites of injury or infection in a wide range of species, leading to the general conception that melanin, and/or its quinone precursors, have anti-infection properties [4]. The synthesis of melanin occurs through enzymes encoded by the tyrosinase gene family, of which dopachrome tautomerase (Dct) is considered to be melanocyte specific [5]. In Atlantic salmon, these genes are expressed in secondary lymphatic organs, where melanin-containing cells, termed melanomacrophages, reside [6]. Expression of the tyrosinase gene family occurs in melanomacrophages during chronic inflammation of Atlantic salmon, indicating a *de novo* melanin synthesis [7]. The observations of atrial melanin deposits in the hearts of CMS-diseased fish [3] made us question if viral cardiac infections might contribute to a general activation of the extracutaneous pigmentary system.

In addition to CMS, marine farmed Atlantic salmon are prone to numerous viral diseases with affinity to the heart, including heart and skeletal muscle inflammation (HSMI, proposed causative agent; piscine reovirus, PRV) [8,9] and pancreas disease (PD, salmon pancreas disease virus, SPDV) [10]. Additionally, infectious salmon anaemia (ISA) caused by the infectious salmon anaemia virus (ISAV) [11] may cause sloughing of cardiac endothelia and severe circulatory disturbances [12,13]. The viral cardiac diseases CMS, HSMI and PD exhibit differences in the immunopathological responses in Atlantic salmon [14].

The aim of this study was to investigate if the melanin deposits in the CMS-associated lesions of the hearts were due to *de novo* synthesis. A secondary aim was to reveal if such melanisation could be part of a general systemic response to common viral infections of Atlantic salmon.

**Figure 1:**
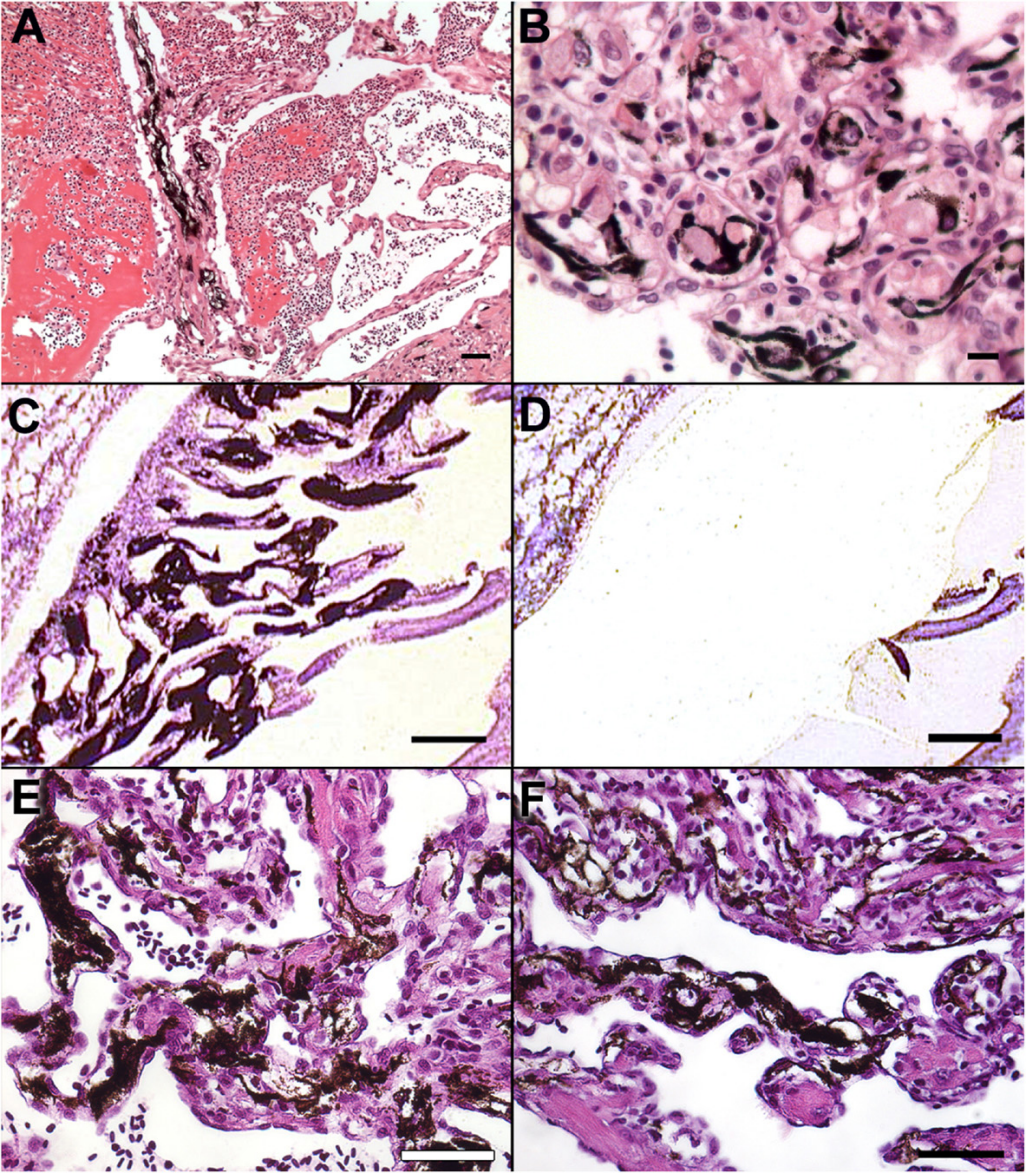
**Pigmented pathological changes in the hearts of CMS-diseased fish. (A-D)** Field outbreak. **(E-F)** Experimental infection study. **(A)** A severely affected area of the atrium where hypertrophic endocardial cells formed tubes filled with heavily pigmented cells interpreted as melanomacrophages. H&E, bar = 50 μm. **(B)** Morphological variation of the melanomacrophages includes elongated, dendritic-shaped cells and rounded macrophage-like cells containing abundant amounts of melanin. H&E, bar = 10 μm. **(C-D)** Microdissection from an area with accumulations of melanomacrophages, before and after laser capture respectively. Haematoxylin, bar = 50 μm. **(E-F)** Melanin deposits in severely affected atrial areas with CMS-associated pathological changes, 24 and 27 wpc, respectively. H&E, bar 50 μm.

Four different production farms of Atlantic salmon were diagnosed with CMS outbreaks by the Norwegian Veterinary Institute. PMCV was detected in all four locations. Formalin fixed paraffin embedded (FFPE) samples from heart and kidney, stained with haematoxylin and eosin (H&E), were included in this study. Histological classification was based on the presence of endocarditis, mononuclear myocarditis, degeneration and necrosis [2], and the findings were graded from 0 to 4 according to defined criteria [3]. Histological analysis of a total of 61 individuals (~15 per farm) revealed 52 fish with severe CMS lesions in the heart, three fish with mild myocarditis (mild CMS) and six fish with no lesions. Among the 52 fish with severe cardiac lesions, 10 had moderate to severe amounts of melanin deposited in the areas of pathological changes. In severely affected areas, mainly in the atrium, hypertrophic endocardial cells formed empty tubes where almost all muscle fibres were replaced by inflammatory cells and heavily pigmented cells interpreted as melanomacrophages (Figure 1A). There was a strong morphological variation of these melanomacrophages throughout the inflamed tissue, from rounded macrophage-like cells to voluminous, dendritic-shaped cells containing abundant amounts of melanin (Figure 1B).

To address possible *de novo* melanin synthesis, samples of pigmented cardiac tissue were analysed for the expression of genes confined to melanin-synthesizing cells. Six individuals with severe atrial melanisation were subjected to laser capture microdissection (LCMD). FFPE-sections, 5 μm thick, were mounted on membrane slides (Molecular Machines and Industries, Zürich – Glattbrugg, Switzerland), dewaxed and counterstained with RNase-free haematoxylin. Sections of pigmented cell accumulations were micro dissected as described previously [7] (Figure 1C-D). From each individual, neighbouring non-pigmented inflamed tissue was sampled for comparison. Total RNA was isolated using a combination of the FFPE-kit and the Nucleo Spin RNA XS (Macherey & Nagel, Düren, Germany). cDNA synthesis was prepared with M-MLV Reverse transcriptase (Promega, Madison, WI, USA) and a combination of oligo(dT) and random hexamer primers. Quantitative real-time PCR was performed and analysed for the expression of tyrosinase (Tyr) and dopachrome tautomerase (Dct) [6]. The expression level was measured with relative quantification using elongation factor 1A_A_ (EF1A_A_) [15] as described previously [7].

**Figure 2:**
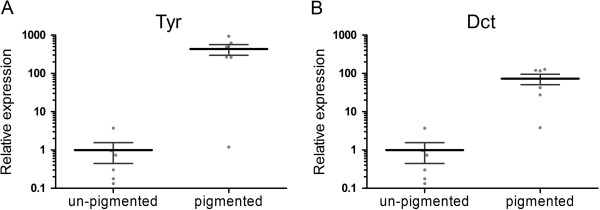
**Laser capture microdissection (LCMD) and RT-qPCR of samples from field outbreak of CMS.** Relative transcription levels of Tyr **(A)** and Dct **(B)** in pigmented and un-pigmented micro dissected tissue samples from the hearts of CMS-diseased Atlantic salmon. The scatter plots show the relative transcription level for each individual. Expression levels in pigmented samples are presented relative to the mean of the un-pigmented, which was given value 1. Error bars represent mean with standard error of the mean (SEM).

The procedure of microdissection enabled us to compare the relative transcription levels of the tyrosinase gene family in the pigmented lesions of diseased fish with respective areas of inflammation without pigmentation. The relative transcription of both Tyr and Dct was significantly up-regulated in inflamed areas containing accumulations of melanomacrophages compared to non-pigmented parts of the inflammatory lesion within the same sample (Figure 2), (paired *t*-test, *p* < 0.05). These results made us able to pinpoint the transcription of the tyrosinase gene family to the pigmented cells, hence strongly suggesting *de novo* melanin production by melanomacrophages.

A disadvantage with field material is that the chronology of events in the disease development is not provided. By including FFPE-samples of heart and kidney from an experimental infection study of CMS [3], a time course study of the disease development could be pursued, including detection of the arrival of inflammatory cells and concurrent presentation of melanisation. The fish had been challenged with heart and kidney homogenate from CMS diseased fish and developed lesions consistent with the disease [2]. Detailed results have been presented previously [3]. In brief, the atrial lesions started as mild to moderate 6 week post challenge (wpc), peaked in severity 12 wpc, and remained at this level for the rest of the study (42 wpc). The most extensive atrial lesion (Grade 4) was recorded in a single fish sampled 24 wpc. By autopsy, sparse amounts of melanin-like deposits were visible in the atrium of one or two challenged fish in every sampling from 24 wpc to termination (42 wpc). Histopathological examinations of these discolorations revealed pigmentation mainly in atrial lesions. The deposits were large in the most severe foci (Figure 1E-F) and resembled the changes described for the field outbreak of CMS (Figure 1A-D). During this study, an unforeseen outbreak of pancreatic necrosis (IPN) was observed in some of the challenged fish [3]. Furthermore, piscine reovirus (PRV) was also detected in the material [16]. The lesions are therefore referred to as CMS-associated pathological changes in PMCV-positive fish co-infected by IPNV and PRV.

Further, to investigate if the extracutaneous pigmentary system is activated as a general response to viral infections, the transcription of Tyr and Dct in whole tissue samples from heart and kidney of experimentally challenged Atlantic salmon was addressed. For this purpose, frozen archive samples of heart and kidney originating from controlled infection studies with CMS, PD, HSMI and ISA was examined (for details see Table 1). The CMS- and PD-material originated from previously described transmission experiments [3,17]. The HSMI-material originated from an experimental transmission study (Rimstad et al., unpublished work). In short, forty fish were injected intraperitoneally with 200 μL organ-homogenate from two individual salmon with HSMI, diagnosed by histology. The organs homogenized were heart, spleen and head kidney diluted 1:10 in Hanks solution. The ISA-material originated from an experimental infection study using a virus bath challenge model (Austbø et al., unpublished work). Briefly, the experimental fish, with an average weight of 113 g, were immersed for 2 h in a total volume of 25 L with a virus concentration at 2.5 × 10^4^ TCID 50 mL-1. Infection by ISAV was confirmed in all challenged fish by real-time PCR. All samples of heart and kidney from the four infection trials of CMS, PD, HSMI and ISA were subjected to RT-qPCR analysis as described previously [7].

**Table 1:** Overview of the material collected from farmed Atlantic salmon subjected to four different controlled experimental virus infections trials and uninfected controls reared in parallel.

**Experiment**	**Sampling time point and number of fish collected**			
*CMS*				
Time of sampling	3 wpc	12 wpc	24 wpc	27 wpc
Infected fish	5 (1*)	5	5	6
Control fish	2	2	3	3
*PD*				
Time of sampling	0 wpc	3 wpc	6 wpc	
Infected fish		10	10	
Control fish	10			
*HSMI*				
Time of sampling	0 wpc	2 wpc	6 wpc	10 wpc
Infected fish		5	5	5
Control fish	5			
*ISA*				
Time of sampling	0 wpc	8 dpc	3 wpc	
Infected fish		5	5	
Control fish	3		2	

Wpc = weeks post challenge. Dpc = days post challenge. (1*) Due to degradation of RNA in the CMS-material, we were only able to include one heart sample from 3 wpc.

**Figure 3:**
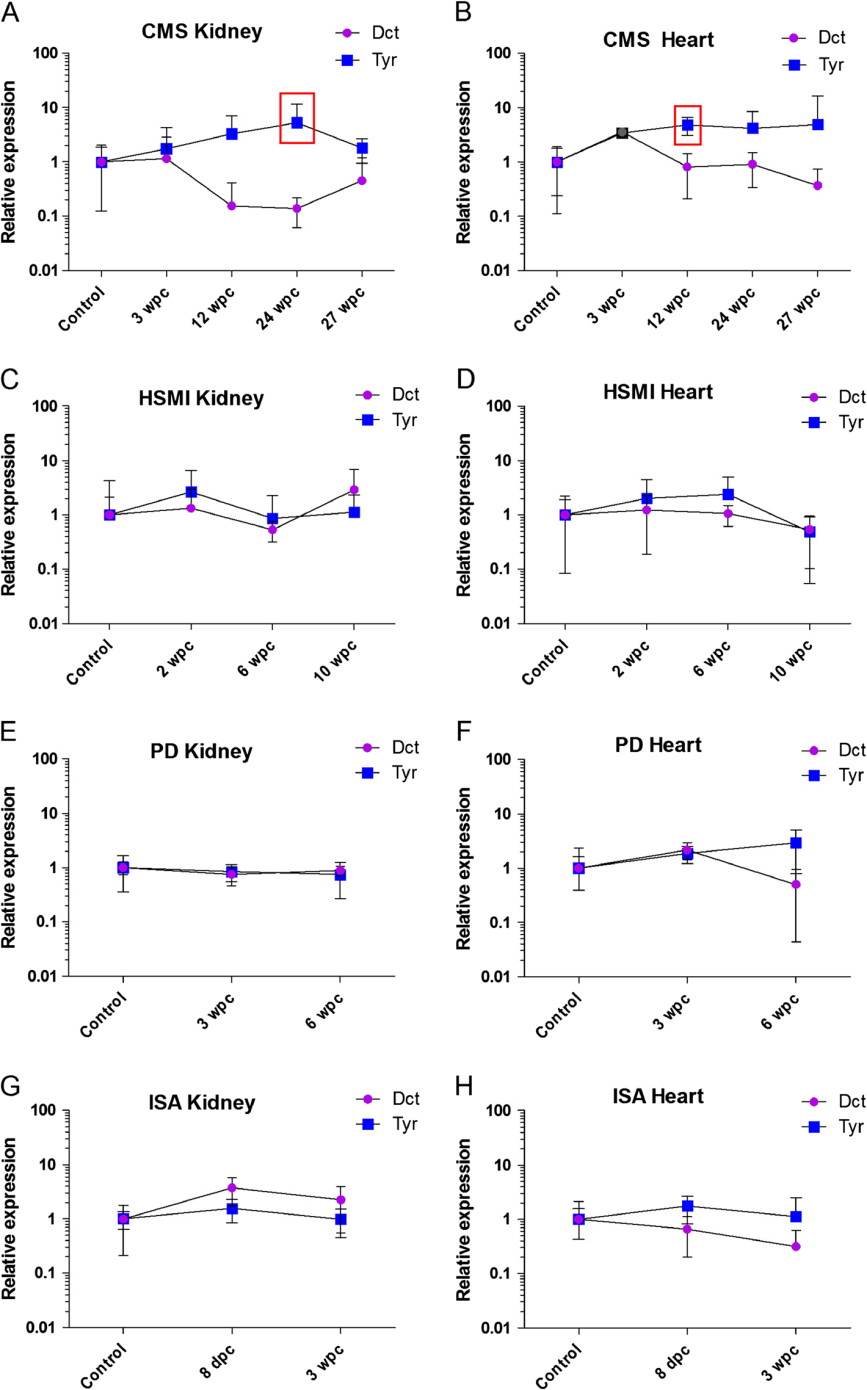
**Relative transcription levels of Tyr and Dct in whole-tissue samples of heart and kidney.** Samples were collected from experimental infection trials of Atlantic salmon with CMS-associated pathological changes (PMCV-positive fish co-infected by IPNV/PRV) **(A-B)**, HSMI (diagnosis set only by histopathology, viral aetiology not confirmed) **(C-D)**, PD **(E-F)** and ISA **(G-H)**. The graph plots show the mean transcript levels for each group as obtained by RT-qPCR analysis. Expression levels are presented relative to the mean of the respective control groups, which were given the value 1. Error bars represent the mean with 95% CI for each group. The level of transcription of the tyrosinase gene family was overall not significantly different in the tissue samples from the infected individuals compared with the non-infected controls, except for Tyr in CMS Kidney 24 wpc, *p* < 0.05 (A) and CMS Heart 12 wpc, *p* < 0.05 (B), as indicated by the red square.

For the whole tissue samples from heart and kidney, RT-qPCR analysis revealed overall no statistical significant differences in the transcription levels of the tyrosinase gene family in the infected fish from the various infection trials when compared with the controls (Figure 3A-H) (unpaired *t*-test and non-parametric Mann–Whitney test). Hence, evidence for a systemic activation of the extracutaneous pigmentary system as a direct response to the viral infections or the cardiac inflammation could not be observed. However, in the experimental study of CMS (PMCV-positive fish co-infected by IPNV and PRV) there was a tendency towards an up-regulation of the transcription of tyrosinase, yet only statistically significant in the kidney at 24 wpc (Figure 3A) and in the hearts at 12 wpc (Figure 3B) but without concordant up-regulation of Dct. Only samples confirmed negative for IPNV were included in the control group. The fish from the different fish farms were euthanized according to regulations for fish in aquaculture issued by the Norwegian Directorate of Fisheries (Forskrift om drift av akvakulturanlegg. § 28. Avlivning av fisk.). The challenge experiments were approved by Norwegian animal welfare authorities (according to FOTS - Forsøksdyrutvalgets tilsyns- og søknadssystem).

## Discussion

In this study, we have investigated pathological pigmentation in cardiac lesions of Atlantic salmon with CMS-associated pathological changes. Pigmented areas of affected hearts collected from field outbreaks of CMS showed expression of genes confined to melanin-synthesizing cells, with an indisputable up-regulation of Tyr- and Dct-transcription compared to neighbouring non-pigmented inflamed areas. These results strongly support the hypothesis of de novo synthesis of melanin at the site of inflammation. Field material may be biased by potential underlying infections that have not been defined. Hence, some reservation must be made in defining PMCV as the exclusive causative agent of these CMS-associated lesions.

During the course of the infection in the experimental study of CMS, melanomacrophages were found to be part of the inflammatory picture only at a late phase in the disease development. The majority of the pigmentation was observed in atrial lesions from 27 wpc, although in some individuals, melanomacrophages were found in the pathological changes at 24 wpc. The atrial changes were the first to occur, reaching a more severe grade compared to those of the spongy ventricle throughout the experiment. In the field, CMS is often observed as sudden death of large fish, due to rupture of the sinus venosus or atrium and resultant cardiac tamponade [2]. However, the pathological changes in the hearts develop over several months, without clinical signs of disease. Terminally, the myocardial lesions reach a severity where the fish can no longer compensate for the compromised function [2]. In fish with PD, the significant lesions are found in the pancreas, heart and skeletal muscle [18]. Nonetheless, fish are able to regenerate myocardial cells [19] and fish sampled in late phase of PD may only have skeletal muscle and pancreatic lesions [20], implicating the possibility of a complete restoration of the heart tissue. The heart is also the predilection organ for PRV in HSMI. Here, cardiac lesions occur earlier after infection than in CMS and persist for many months before being gradually reduced, implying that the fish may be able to control the infection and concordantly compensate the tissue damage [21]. Furthermore, the cardiac changes of HSMI are less degenerative compared to CMS and PD [22]. We suspect that the accumulation of melanin arises in areas of pathological changes too damaged to be restored, as would be the case for the heart in CMS. Thus, melanin seems to play a role particularly in the most severely damaged parts of the chronic CMS-lesions.

The enzymatic intermediates of melanin synthesis are known to modulate reactive oxygen species (ROS) through their antioxidative properties [23]. ROS can harm cellular constituents (oxidative stress), thereby enhancing the need of free radical trapping [24]. This may be the case for the melanomacrophages participating in the chronic inflammatory changes in the hearts of the CMS-diseased fish. Chronic inflammation may be stimulated by damaged and dying cells [25] and antigen-presenting cells may be activated by alarm signals from stressed or damaged tissues [26]. Concordantly, teleost melanomacrophages may be attracted and stimulated by the reparative processes following the necrosis caused by the CMS-virus.

Melanin and melanogenesis have been considered to be related to innate immunity in a range of species [4,27,28] and melanomacrophages may express MHC class II receptors when participating in chronic inflammation [7,29]. In a previous in vitro study of viral infection and melanogenesis, the viral antigen stimulation seemed to hinder the induction of the tyrosinase gene family, at least in the early phase of infection [30]. However, in vitro studies may not be reflecting real life and it is not known if virus infection may induce up-regulation of the tyrosinase gene family in the extracutaneous pigmentary system of Atlantic salmon in vivo. By monitoring the expression pattern of Tyr and Dct in the heart and kidney throughout the course of the virus infection experiments, we addressed this possibility. In the experimental study of CMS (PMCV-positive fish co-infected by IPNV and PRV) there was a tendency towards an up-regulation of the transcription of Tyr, yet only statistically significant in the kidney at 24 wpc and in the hearts at 12 wpc. However, this trend could not be observed in the transcription of Dct. When analysing whole blocks of tissue, small differences in transcription levels may be obscured. This could be the reason for the diverging result of RT-qPCR of whole tissue in the experimental trial of CMS and the more precisely micro dissected tissue in the field material. No significant up-regulation of the tyrosinase gene family was found in PD, HSMI or ISA. Taken together, a collaborative role of the pigmentary system as part of the innate immune defence towards viral infections could not be established based on results in this study. This is in line with our previous in vitro studies of viral infection and melanogenesis [30]. Teleost fish possess melanin-producing leukocytes, but in contrast to insects, which do not harbour an adaptive immune system as characterized by the presence of molecules of the immunoglobulin superfamily, melanin seems to have a subordinate role in defence actions. Hence, indicating that pigment-production plays a more indirect role in viral cardiac inflammatory reactions in Atlantic salmon and not as a response to the virus infection *per se*. Taking the present data into account, we suggest that melanin production in chronically inflamed cardiac lesions of CMS-diseased fish serve as a protective mechanism against oxidation during cell necrosis followed by repair by scarring in tissue too damaged to be restored.

## Abbreviations

CMS: Cardiomyopathy syndrome; PMCV: Piscine myocarditis virus; HSMI: Heart and skeletal muscle inflammation; PRV: Piscine reovirus; PD: Pancreas disease; SPDV: Salmon pancreas disease virus; ISA: Infectious salmon anaemia; ISAV: Infectious salmon anaemia virus; FFPE: Formalin fixed paraffin embedded; LCMD: Laser capture microdissection; RT-qPCR: Quantitative real-time reverse transcriptase polymerase chain reaction; Tyr: Tyrosinase; Dct: Dopachrome tautomerase.

## Competing interests

The authors declare that they have no competing interests.

## Authors’ contributions

HL designed the study, performed experiments, interpreted results and wrote the manuscript. LA provided the ISA- and PD-material, interpreted results and edited the manuscript. CF provided the CMS-material from the infection trial, performed histology, interpreted results and co-wrote the manuscript. MA provided the CMS-material from the field outbreak and performed histology. TT provided the CMS-material from the infection trial. ER provided the PD- and HSMI-material. KF provided ISA-material. EOK supervised the study, interpreted results and edited the manuscript. All authors read and commented on the manuscript.

## References

[B1] Haugland Ø Mikalsen AB Nilsen P Lindmo K Thu BJ Eliassen TM Roos N Rode M Evensen Ø Cardiomyopathy syndrome of atlantic salmon (*Salmo salar* L.) is caused by a double-stranded RNA virus of the totiviridae family J Virol 2011 85 5275 5286 10.1128/JVI.02154-10 21411528 PMC3094960

[B2] Ferguson HW Poppe T Speare DJ Cardiomyopathy in farmed Norwegian salmon Dis Aquat Organ 1990 8 225 231

[B3] Fritsvold C Kongtorp RT Taksdal T Ørpetveit I Heum M Poppe TT Experimental transmission of cardiomyopathy syndrome (CMS) in Atlantic salmon *Salmo salar* Dis Aquat Organ 2009 87 225 234 20099415 10.3354/dao02123

[B4] Mackintosh JA The antimicrobial properties of melanocytes, melanosomes and melanin and the evolution of black skin J Theor Biol 2001 211 101 113 10.1006/jtbi.2001.2331 11419954

[B5] Kelsh RN Schmid B Eisen JS Genetic analysis of melanophore development in zebrafish embryos Dev Biol 2000 225 277 293 10.1006/dbio.2000.9840 10985850

[B6] Thorsen J Høyheim B Koppang EO Isolation of the Atlantic salmon tyrosinase gene family reveals heterogenous transcripts in a leukocyte cell line Pigment Cell Res 2006 19 327 336 10.1111/j.1600-0749.2006.00319.x 16827751

[B7] Larsen HA Austbø L Mørkøre T Thorsen J Hordvik I Fischer U Jirillo E Rimstad E Koppang EO Pigment-producing granulomatous myopathy in Atlantic salmon: a novel inflammatory response Fish Shellfish Immunol 2012 33 277 285 10.1016/j.fsi.2012.05.012 22634154

[B8] Finstad Ø Falk K Løvoll M Evensen Ø Rimstad E Immunohistochemical detection of piscine reovirus (PRV) in hearts of Atlantic salmon coincide with the course of heart and skeletal muscle inflammation (HSMI) Vet Res 2012 43 27 10.1186/1297-9716-43-27 22486941 PMC3384478

[B9] Palacios G Løvoll M Tengs T Hornig M Hutchison S Hui J Kongtorp RT Savji N Bussetti AV Solovyov A Kristoffersen AB Celone C Street C Trifonov V Hirschberg DL Rabadan R Egholm M Rimstad E Lipkin WI Heart and skeletal muscle inflammation of farmed salmon is associated with infection with a novel reovirus PLoS One 2010 5 e11487 10.1371/journal.pone.0011487 20634888 PMC2901333

[B10] Nelson RT McLoughlin MF Rowley HM Platten MA McCormick JI Isolation of a toga-like virus from farmed Atlantic salmon *Salmo salar* with pancreas disease Dis Aquat Organ 1995 22 25 32

[B11] Dannevig BH Falk K Namork E Isolation of the causal virus of infectious salmon anaemia (ISA) in a long-term cell line from Atlantic salmon head kidney J Gen Virol 1995 76 1353 1359 10.1099/0022-1317-76-6-1353 7782764

[B12] Roberts RJ Roberts RJ The pathophysiology and systematic pathology of teleosts Fish Pathology 2012 4 West Sussex: Wiley-Blackwell 62 143

[B13] Thorud K Djupvik HO Infectious anaemia in Atlantic salmon (*Salmo salar* L.) Bull Eur Ass Fish Pathol 1988 8 109 111

[B14] Yousaf MN Koppang EO Skjødt K Köllner B Hordvik I Zou J Secombes C Powell MD Cardiac pathological changes of Atlantic salmon (*Salmo salar* L.) affected with heart and skeletal muscle inflammation (HSMI) Fish Shellfish Immunol 2012 33 305 315 10.1016/j.fsi.2012.05.008 22609767

[B15] Olsvik PA Lie KK Jordal AE Nilsen TO Hordvik I Evaluation of potential reference genes in real-time RT-PCR studies of Atlantic salmon BMC Mol Biol 2005 6 21 10.1186/1471-2199-6-21 16293192 PMC1314898

[B16] Wiik-Nielsen J Løvoll M Fritsvold C Kristoffersen AB Haugland Ø Hordvik I Aamelfot M Jirillo E Koppang EO Grove S Characterization of myocardial lesions associated with cardiomyopathy syndrome in Atlantic salmon, *Salmo salar* L., using laser capture microdissection J Fish Dis 2012 35 907 916 10.1111/j.1365-2761.2012.01431.x 22913811

[B17] Grove S Austbø L Hodneland K Frost P Løvoll M McLoughlin M Thim HL Braaen S König M Syed M Jørgensen JB Rimstad E Immune parameters correlating with reduced susceptibility to pancreas disease in experimentally challenged Atlantic salmon (*Salmo salar*) Fish Shellfish Immunol 2013 34 789 798 10.1016/j.fsi.2012.12.014 23306092

[B18] Rodger HD Turnbull T Richards RH Myopathy and pancreas disease in salmon: a retrospective study in Scotland Vet Rec 1994 135 234 235 10.1136/vr.135.10.234 7801443

[B19] Poss KD Wilson LG Keating MT Heart regeneration in zebrafish Science 2002 298 2188 2190 10.1126/science.1077857 12481136

[B20] McLoughlin MF Graham DA Alphavirus infections in salmonids–a review J Fish Dis 2007 30 511 531 10.1111/j.1365-2761.2007.00848.x 17718707

[B21] Kongtorp RT Halse M Taksdal T Falk K Longitudinal study of a natural outbreak of heart and skeletal muscle inflammation in Atlantic salmon, *Salmo salar* L J Fish Dis 2006 29 233 244 10.1111/j.1365-2761.2006.00710.x 16635063

[B22] Kongtorp RT Taksdal T Lyngoy A Pathology of heart and skeletal muscle inflammation (HSMI) in farmed Atlantic salmon *Salmo salar* Dis Aquat Organ 2004 59 217 224 15264718 10.3354/dao059217

[B23] Sichel G Biosynthesis and function of melanins in hepatic pigmentary system Pigment Cell Res 1988 1 250 258 10.1111/j.1600-0749.1988.tb00423.x 3148917

[B24] Lushchak VI Environmentally induced oxidative stress in aquatic animals Aquat Toxicol 2011 101 13 30 10.1016/j.aquatox.2010.10.006 21074869

[B25] Hamilton JA Nondisposable materials, chronic inflammation, and adjuvant action J Leukoc Biol 2003 73 702 712 10.1189/jlb.0103037 12773502

[B26] Matzinger P The danger model: a renewed sense of self Science 2002 296 301 305 10.1126/science.1071059 11951032

[B27] Agius C Roberts RJ Melano-macrophage centres and their role in fish pathology J Fish Dis 2003 26 499 509 10.1046/j.1365-2761.2003.00485.x 14575368

[B28] Plonka PM Passeron T Brenner M Tobin DJ Shibahara S Thomas A Slominski A Kadekaro AL Hershkovitz D Peters E Nordlund JJ Abdel-Malek Z Takeda K Paus R Ortonne JP Hearing VJ Schallreuter KU What are melanocytes really doing all day long…? Exp Dermatol 2009 18 799 819 10.1111/j.1600-0625.2009.00912.x 19659579 PMC2792575

[B29] Haugarvoll E Bjerkås I Szabo NJ Satoh M Koppang EO Manifestations of systemic autoimmunity in vaccinated salmon Vaccine 2010 28 4961 4969 10.1016/j.vaccine.2010.05.032 20553770

[B30] Larsen HA Austbø L König M Sørum H Rimstad E Koppang EO Transcription of the tyrosinase gene family in an Atlantic salmon leukocyte cell line (SHK-1) is influenced by temperature, but not by virus infection or bacterin stimulation Dev Comp Immunol 2013 41 50 58 10.1016/j.dci.2013.03.019 23562574

